# MiR146a modulates chondrogenesis of bone marrow mesenchymal stem cells by modulating Lsm11 expression

**DOI:** 10.1152/ajpcell.00460.2022

**Published:** 2023-03-20

**Authors:** Yuhang Liu, Xudong Zhang, Xiaodong Chen, Bingjun Zhang, Liming Dai, Chenglong Wang, Yang Li, Xiaoling Zhang

**Affiliations:** ^1^Department of Orthopedic Surgery, Xin Hua Hospital Affiliated to Shanghai Jiao Tong University School of Medicine, Shanghai, China; ^2^Department of Pathology, The University of Texas MD Anderson Cancer Center, Houston, Texas, United States; ^3^National Facility for Translational Medicine (Shanghai), Shanghai, China

**Keywords:** bone marrow mesenchymal stem cells, chondrogenesis, Lsm11, microRNA146a, osteoarthritis

## Abstract

MicroRNAs play a critical role in bone marrow mesenchymal stem cell (MSC) chondrogenesis and regulate the progression of joint regeneration in osteoarthritis. Our previous research confirmed that miR146a relieves osteoarthritis by modulating cartilage homeostasis. However, few studies have revealed the relationship between miR146a and the chondrogenesis of MSCs, and the exact mechanisms remain unclear. This study aimed to determine the function of miR146a in the chondrogenic differentiation of MSCs and the potential mechanisms involved. MiR146a expression increased during chondrogenesis. MiR146a knockout (KO) led to the increased chondrogenesis of MSCs compared to that in wild-type (WT) MSCs, whereas the overexpression of miR146a by mimics resulted in the decreased chondrogenesis of MSCs, as determined by the mRNA expression of collagen, type II, alpha 1 (COL2A1), aggrecan, cartilage oligomeric matrix protein (COMP), and matrix metallopeptidase 13 (MMP13). Furthermore, cartilage defects could be treated better when injected with spheres induced from miR146aKO MSCs than from WT MSCs, indicating that miR146a inhibits chondrogenesis in vivo. In addition, based on miRNA-mRNA prediction analysis and a dual-luciferase reporter assay, we observed that the deletion of miR146a led to the increased expression of Lsm11 during chondrogenesis and demonstrated that miR146a targeted Lsm11 by binding to its 3′-untranslated region (UTR) and inhibited its translation. The inhibition of Lsm11 by silencing RNA (siRNA) reversed the increased ability of chondrogenesis by knocking out miR146a both in vivo and in vitro, suggesting that miR146a inhibits chondrogenesis by directly inhibiting Lsm11 in MSCs, which may be a novel target for treating osteoarthritis.

## Graphical abstract

**Figure:**
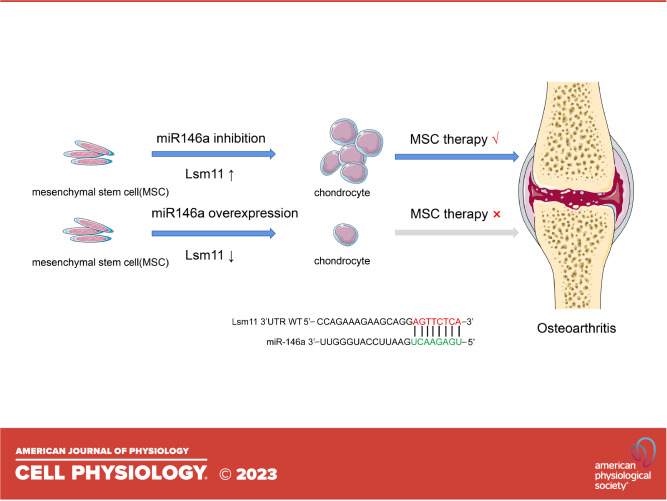

## INTRODUCTION

Osteoarthritis (OA) is a common degenerative disease of the whole joint that affects millions of people worldwide. It is characterized by cartilage degradation, subchondral bone remodeling, chronic synovitis, osteophyte formation, and meniscal alterations (1–3). OA has been proven to occur in 20% of individuals aged >65 yr (3), and the primary pathology of OA includes cellular senescence (4), age-related inflammation, and metabolic alterations in functional cells (5). Although increasing investigations have revealed the complex mechanisms of OA, its treatment in the clinic remains unsatisfactory, and there are no effective methods to control the progression of OA and delay or reverse cartilage defects owing to the restricted self-regenerative capacity of chondrocytes. However, growing evidence indicates that chondrogenesis of bone marrow mesenchymal stem cells might be a novel functional target to treat OA, since cartilage formation from injected mesenchymal stem cells (MSCs) in vivo could supply normal chondrocytes to a great extent (6). Thus it is essential to reveal the exact mechanisms of chondrogenesis in MSCs, with the aim of finding more effective OA treatment methods.

Chondrogenesis in MSCs is regulated by distinct mechanisms, in which microRNAs (miRNAs) play an essential role by targeting genes by binding to the 3′-untranslated region (UTR) and regulating gene expression (7). A series of miRNAs, including miR26, miR140, miR145, and miR337, control the chondrogenesis of MSCs and the progression of OA (8). We have previously shown that miR146a can aggravate cartilage degeneration and accelerate OA progression by targeting Camk2d and Ppp3r2 (9). As previously reported, miR146a functions as a vital regulator of inflammation, the immune system, metastasis, and disease progression (10–12). However, the role of miR146a in chondrogenesis remains unclear. We aimed to elucidate the exact mechanisms by which miR146a modulates osteoarthritis, not only in chondrocytes but also in mesenchymal stem cells.

In this study, we determined the function of miR146a in the chondrogenesis of murine MSCs using miR146a knockout (miR146aKO) mice and miR146a mimic and found that miR146a serves as an inhibitor of chondrogenesis in vivo and in vitro. Furthermore, we confirmed Lsm11 as a direct target downstream of miR146a based on the prediction of bioinformatics software (TargetScan and miRDB) and luciferase reporter assays. Lsm11 is a U7 snRNP protein that is necessary for U7 snRNP localization and histone pre-mRNA processing (13). Few studies have revealed the role of Lsm11 in chondrogenesis and OA. Thus we hypothesized that miR146a plays an important role in modulating chondrogenesis by regulating Lsm11 expression. Inhibition of Lsm11 considerably reversed the increased chondrogenesis of miR146a KO MSCs, indicating that miR146a inhibits chondrogenesis by targeting and inhibiting Lsm11. Finally, we aimed to confirm the function of miR146a in vivo by transferring cartilage spheres. To conclude, inhibiting miR146a might be a novel target to enhance transplanted MSCs in the treatment of OA.

## MATERIALS AND METHODS

### Animals

C57BL/6J mice were purchased from the Shanghai Laboratory Animal Center (Shanghai, China) and the Chinese Academy of Sciences (Shanghai, China). MiR146a homozygous knockout mice (designated as miR146aKO) were on a C57BL/6 background and were first obtained from Jackson Laboratory (Bar Harbor, ME), which were heterozygous and intercrossed in our animal facility. After a few generations, we detected miR146aKO mice in the offspring by screening for genotype. Sequences of the primer for identification of the genotype were as follows: forward primer 5′- GACGAGCTGCTTCAAGTTCC-3′ and reverse primer 5′- ACCAGCAGTCCTCT- TGATGC-3′. All animal experiments were performed in a pathogen-free animal facility at Shanghai Jiaotong University of Medicine, Xinhua Hospital and were approved by the Institutional Ethics Committee of Xinhua Hospital affiliated with Shanghai Jiaotong University School of Medicine. The animal allowance number of Shanghai Jiaotong University of Medicine, Xinhua Hospital is SYXK-2018-0038.

### Experimental Model of Mice

A surgical model of cartilage defect was induced in 4-mo-old male WT mice through drill penetration at the trochlea of the femur. After penetration, cartilage spheres induced by MSCs were directly placed into the area of the defect for further detection of chondrogenesis. Four weeks after surgery, the knee joints of mice were harvested for histological investigations. The surgical model was established in mice under general anesthesia, and the mice were euthanized via cervical dislocation.

### Isolation of Bone Marrow MSCs and Cell Culture

Mouse primary bone marrow MSCs were isolated from the femur and tibia of 4-mo-old mice by adherence method. MSCs were cultured in DMEM supplemented with 10% FBS purchased from Gibco (Thermo Fisher Scientific, Waltham, MA) and 1% penicillin-streptomycin purchased from Hyclone (GE Healthcare Life Sciences, Logan, UT). MSCs at passages 1–2 were prepared for the experiments. HEK293T cells were obtained from ATCC (Manassas, VA).

### Chondrogenic Differentiation

Chondrogenesis of murine primary bone marrow MSCs was induced in chondrogenic medium, including 48 mL of DMEM; 500 µL of 1% penicillin-streptomycin; 500 µL of insulin, human transferrin, and sodium selenite (ITS); 50 µL of linoleic acid (4.7 µg/mL); 5 µL of dexamethasone (100 nM); 500 µL of BSA (0.5 mg/mL); 500 µL of vitamin C (37.5 µg/mL); and 50 µL of proline (40 µg/mL). The medium contained no FBS to limit MSC proliferation. Cartilage spheres were induced by droplets (10 µL) of suspended MSCs in 500 µL chondrogenic medium in 24-well plates for 21 days. MSCs were collected at different time points for chondrogenic induction according to the design.

### Quantitative PCR

Mouse primary bone marrow MSCs were cultured and rinsed with PBS, and total RNA was extracted by transfer to TRIzol reagent (Invitrogen, Waltham, MA) from 6-well plates and then homogenized at high speed on ice. DNase I (Sigma-Aldrich, St. Louis, MO) was added to the extracted mRNA to remove genomic DNA. The quantification of mRNA was performed and calculated through Nanodrop 2000 (Thermo Fisher Scientific). mRNA was reverse transcribed into complementary DNA (cDNA) in each experimental and control group using the PrimeScript RT Master Mix Kit (Takara Bio Inc., Dalian, China). Then cDNA was tested by PCR via the SYBR Premix Ex Taq Kit (RR420a; Takara, Tokyo, Japan). To normalize the mRNA expression, the level of the housekeeping gene GAPDH served as a control. Expression of miR-146a and miR140 was assayed with specific TaqMan kits from Applied Biosystems (Thermo Fisher Scientific) and U6 snRNA was used to normalize the expression. Quantitative PCR (qPCR) primers for the genes and forward (F) and reverse (R) primer sequences were as follows: AGGRECAN-F: 5′- GTGGAGCCGTGTTTCCAAG-3′ and AGGRECAN-R:5′- AGATGCTGTTGACTCGAACCT-3′; cartilage oligomeric matrix protein (COMP)-F: 5′- ACTGCCTGCGTTCTAGTGC-3′ and COMP-R: 5′- CGCCGCATTAGTCTCCTGAA-3′; collagen, type II, alpha 1 (COL2A1)-F: 5′- ACGAGGCAGACAGTACCTTG-3′ and COL2A1-R: 5′- CAGCCCTGGTTGGGATCAAT-3′; SOX9-F: 5′- TCAGCAAGACTCTGGGCAAG-3′ and SOX9-R: 5′- TCCGTTCTTCACCGACTTCC-3′; matrix metallopeptidase 13 (MMP13)-F: 5′- TTGGCTTAGAGGTGACTGGC-3′ and MMP13-R: 5′- CCACATCAGGCACTCCACAT-3′; GAPDH-F: 5′- TGACCTCAACTACATGGTCTACA-3′ and GAPDH-R: 5′- CTTCCCATTCTCGGCCTTG-3′; Slc10a3-F: 5′- CCACACAAGTGGTCACTATTTGA-3′ and Slc10a3-R: 5′- TCCAGTGTATTGGCTAGAGATCA-3′; TDRKH-F: 5′- TCCACTGAACGAACTTCATGG-3′ and TDRKH-R: 5′- CGACGATACAGAATGTAGGCAAC-3′; FBXW2-F: 5′- GGACATAGTGCCAGAGTGTATGC-3′ and FBXW2-R: 5′- GCCCTGTGCTTACATCCCA-3′; and BCORL1-F: 5′- CCCCAGCCCCAATCTTTACTC-3′ and BCORL1-R: 5′- AAAACACACTCGACTCAAACTGA-3′; SBSPON-F:5′- CTGCTGCTTCGACTACGACAG-3′ and SBSPON-R: 5′- CCTCCGTTTAGTGGTTCCTGA-3′; Appl1-F: 5′- AGCCAGTGACCCTTTATATCTGC-3′ and Appl1-R: 5′- AGGTATCCAGCCTTTCGGGTT-3′; Lsm11-F: 5′- CACCCCGCAATGTGCTTAC-3′ and Lsm11-R: 5′- GGCCATATTCCAGAACTTGTCG-3′; and MiR-146a-5p : 5′- GCAGTGAGAACTGAATTCCA-3′.

### Western Blot Analysis

The cells were cultured and disposed in lysis buffer with 1 mM PMSF and subjected to SDS-PAGE. Nitrocellulose membranes with transferred protein were blocked in BSA before incubation with primary antibodies. Primary antibodies include aggrecan (1:1,000, A11691; Abcam, Cambridge, UK), matrix metallopeptidase 13 (MMP13) (1:1,000, ab39012; Abcam), and GAPDH (1:3,000, 5174 T; Abcam). Secondary antibodies (7074P2) were purchased from Cell Signaling Technology (Danvers, MA). Chemiluminescent detection was performed using the ECL Enhanced Luminescent Kit (E412-02; Vazyme, Nanjing, China). Quantification of Western blots was performed using Image J by measuring the gray value of each blot and then calculating the *P* value from the *t* test.

### 3′-UTR Cloning and Luciferase Reporter Assay

The 3**′**-UTR of Lsm11 containing the target sequence of miR-146a was amplified by PCR and inserted between the XhoI and NotI sites downstream of Renilla luciferase gene in psiCHECK-2 vector (Promega, Sunnyvale, CA). The QuikChange Lightning site-Directed Mutagensis Kit (Agilent Technologies, Santa Clara, CA) was used to mutate the binding site of miR-146a. For the luciferase assay, 293 T cells were cultured in 96-well plates with 3,000 cells per well. Twenty nanograms luciferase construction and 80 ng miR-146a precursor mimic or mimic control plasmid were used in each well for the 24-h transfection. After 48 h of transfection, we lysed the cells and measured the activity of the luciferase on the luminometer using the dual luciferase reporter assay system (Promega). Experiments were independently repeated at least three times.

### Plasmids and siRNAs

To identify the direct targets of miR146a, full-length mRNA encoding the mouse U7 snRNP-specific Sm-like protein LSM11 (Lsm11; NCBI gene ID 72290) was cloned into the pEGFP-N1 vector (Takara Bio USA, Inc., Mountain View, CA). siRNAs targeting mouse Lsm11 were purchased from GenePharma Co. Ltd. (Suzhou, China). Bone marrow MSCs were transfected using Lipofectamine 2000 (Invitrogen) in serum-free standard cell culture medium. The sequence of siRNA targeting Lsm11 was as follows: sense (5′–3′): GGAAGCGGAUUCCAAAUCUTT and antisense (5′–3′): AGAUUUGGAAUCCGCUUCCTT.

### Immunohistochemistry and Pathological Staining

Collagen, type II, alpha 1 (COL2A1), aggrecan, and MMP13 were examined by immunohistochemistry. Paraffin sections of the cartilage spheres were deparaffinized, rehydrated, pretreated with pepsin for 30 min at 37°C, and then incubated with 3% H_2_O_2_ in methanol solution. After flushing with PBS, the sections were blocked with BSA for 1 h at 26°C and then incubated with primary antibodies at 4°C overnight. The sections were then incubated for 15 min with the secondary antibody supplied in the HRP-Polymer Anti-Rabbit IHC Kit (KIT-5005; MaxVision, Shenzhen, China) and colored by substrates from the DAB Plus Kit (DAB-2031) for 10 min. The antibody of COL2A1 was purchased from Abcam (ab34712), the antibody of aggrecan was purchased from ABclonal (A11691; Wuhan, China), and the antibody of MMP13 was purchased from Abcam (ab39012). Knee histology images were obtained after safranine O, fast green, and Alcian blue staining. Safranine O and fast green staining were carried out for 5 min for each section to examine the cartilage in the joints. Alcian blue staining was used to stain the sections for 30 min. Images were obtained using a microscope from Zeiss and were analyzed using iViewer.

### Statistical Analysis

Data are expressed as the mean ± SD. The Student’s *t* test was used for statistical comparison between groups. Statistical significance was set at *P* < 0.05. For each experiment, at least three replicates were used.

## RESULTS

### Expression of miR146a Increased during Chondrogenesis of Murine MSCs

**Figure 1.**
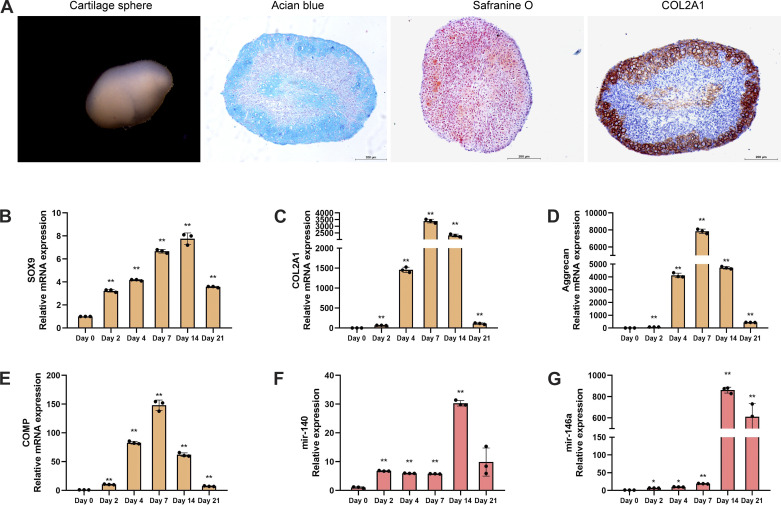
Expression of chondrogenic-related genes and miR146a in the chondrogenesis of murine mesenchymal stem cells (MSCs). MSCs were isolated from wild-type (WT) mice and cultured in chondrogenic medium for 21 days in vitro. *A*: representative images of Alcian blue staining, safranine O staining, and the immunohistochemistry of collagen, type II, alpha 1 (COL2A1) at 21 days after the induction of chondrogenesis in WT MSCs. Scale bar = 200 µm. *B*–*E*: mRNA expression of chondrogenic markers SOX9, COL2A1, aggrecan, and cartilage oligomeric matrix protein (COMP) determined by quantitive PCR during chondrogenesis at 0, 2, 4, 7, 14, and 21 days. *F*: relative mRNA expression of miR140 during chondrogenesis. *G*: relative mRNA expression of miR146a during chondrogenesis. Data represent the mean ± SD of three independent experiments using samples from three different donors. *P < 0.05, **P < 0.01.

To examine the function of miR146a in chondrogenesis, we freshly isolated MSCs from 4-mo-old wild-type (WT) mice and induced them into chondrocytes in serum-free chondrogenic medium in vitro. After 21 days of induction, MSCs differentiated into chondrocytes and assembled to form cartilage spheres (Fig. 1*A*). Pathological staining of cartilage spheres also proved successful chondrogenesis of MSCs in vitro, as examined by Alcian blue staining, safranine O staining, and immunohistochemistry of collagen, type II, alpha 1 (COL2A1) (Fig. 1*A*). Increased expression of the chondrogenic markers SOX9, COL2A1, aggrecan, and cartilage oligomeric matrix protein (COMP) obviously confirmed chondrogenic differentiation 21 days after the induction of chondrogenesis (Fig. 1, *B*–*E*). In addition, the relative mRNA expression of miR140 increased during chondrogenesis, which is a positive control for successful chondrogenic induction (Fig. 1*F*) (14). During chondrogenesis, we observed a significant increase in the expression of miR146a, suggesting that it may play a specific role in the chondrogenesis of MSCs (Fig. 1*G*).

### MiR146aKO MSCs Exhibited Greater Chondrogenic Ability than WT MSCs

**Figure 2.**
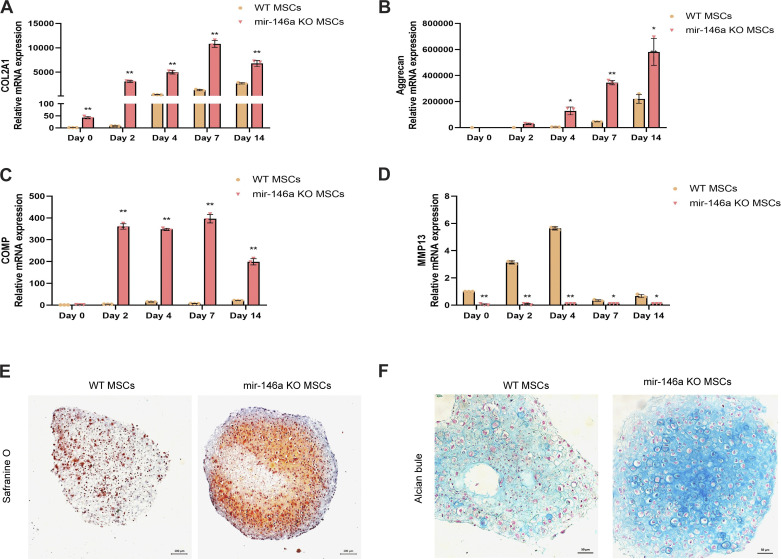
MiR146a knockout led to increased chondrogenesis of murine mesenchymal stem cells (MSCs). MSCs from 4-mo-old wild-type (WT) and miR146a knockout (miR146aKO) mice were isolated and cultured in a chondrogenic medium in vitro. *A*–*D*: mRNA expression of collagen, type II, alpha 1 (COL2A1), aggrecan, cartilage oligomeric matrix protein (COMP), and matrix metallopeptidase 13 (MMP13) in WT and miR146aKO MSCs determined by quantitative PCR during chondrogenesis at 0, 2, 4, 7, and 14 days. *E*: representative images of safranine O-stained WT MSCs and miR146aKO MSCs-derived spheres for 14 days. Scale bar = 100 µm. *F*: representative images of Alcian blue-stained WT MSCs and miR146aKO MSCs-derived spheres after 14 days. Scale bar = 50 µm. Means ± SD are indicated for 3 independent experiments using samples from 3 different donors. *P < 0.05, **P < 0.01.

To investigate whether miR146a could regulate MSC chondrogenesis, we purchased mice with miR146a deletions, termed miR146KO mice. MSCs from 4-mo-old miR146aKO and WT mice were collected and cultured in vitro for chondrogenic induction. During the 14 days of MSC chondrogenesis, the deletion of miR146a led to the enhanced mRNA expression of COL2A1, aggrecan, and COMP, and decreased mRNA expression of matrix metallopeptidase 13 (MMP13), suggesting that miR146a could inhibit the chondrogenic differentiation of MSCs (Fig. 2, *A*–*D*). To further confirm the function of miR146a in chondrogenesis, we induced cartilage spheres in miR146aKO and WT MSCs. The spheres induced from miR146aKO MSCs showed an obvious increase in cartilage formation compared with that in WT MSCs, as confirmed by safranin O and Alcian blue staining, suggesting a vital inhibitory role of miR146a in MSC chondrogenesis (Fig. 2, *E* and *F*).

### Overexpression of miR146a Decreased Chondrogenesis of MSCs

**Figure 3.**
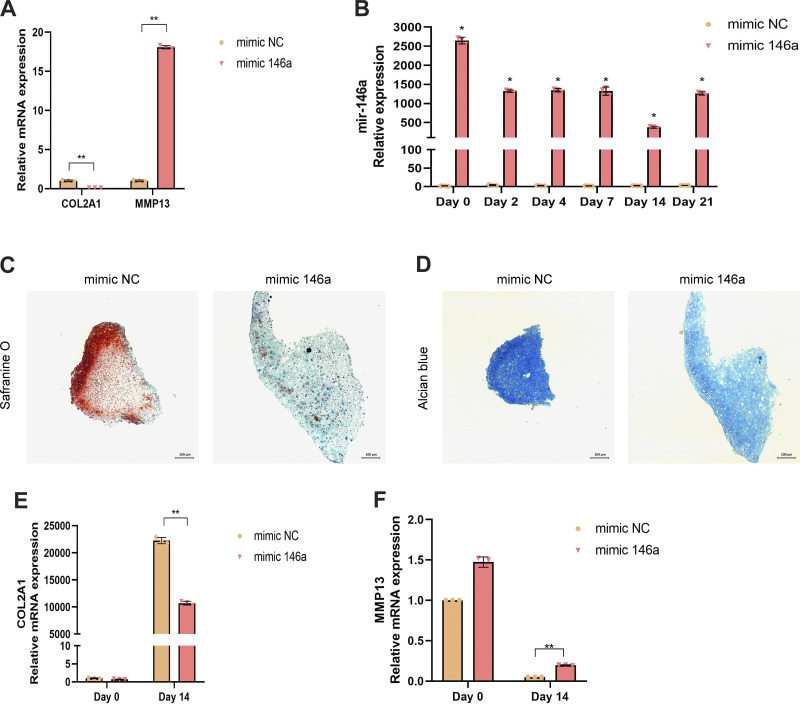
Overexpression of miR146a decreased chondrogenesis of murine mesenchymal stem cells (MSCs). Wild-type (WT) MSCs were transfected with mimic 146a and induced into chondrocytes. *A*: mRNA expression of collagen, type II, alpha 1 (COL2A1) and matrix metallopeptidase 13 (MMP13) in MSCs treated with the mimic negative control (NC) and mimic 146a on *day 14*. *B*: mRNA expression of miR146a was determined by quantitative PCR (qPCR) at 0, 2, 4, 7, 14, and 21 days of chondrogenesis in MSCs treated with mimic NC and mimic 146a. *C*: representative image of safranine O staining of spheres derived from MSCs treated with mimic NC and mimic 146a for 14 days. Scale bar = 100 µm. *D*: representative image of Alcian blue-stained spheres derived from MSCs treated with mimic NC and mimic 146a for 14 days. Scale bar = 100 µm. *E*: mRNA expression of COL2A1 was determined by qPCR after 0 and 14 days of chondrogenesis in MSCs treated with mimic NC and mimic 146a. *F*: mRNA expression of MMP13 was determined by qPCR after 0 and 14 days of chondrogenesis in MSCs treated with mimic NC and mimic 146a. Data represent the mean ± SD of 3 independent experiments using samples from 3 different donors. *P < 0.05, **P < 0.01.

To further confirm the inhibitory role of miR146a on chondrogenesis, WT MSCs were treated with an miR146a mimic to achieve overexpression in vitro before the induction of chondrocyte differentiation. After 14 days of transfection with the miR146a mimic (mimic 146a), the expression of COL2A1 decreased and the expression of MMP13 increased in MSCs compared to MSCs treated with the mimic negative control (mimic NC) (Fig. 3*A*). The mRNA expression of miR146a increased significantly during the chondrogenesis process when treated with the mimic 146a compared to the mimic NC, indicating the successful overexpression of miR146a by the mimic (Fig. 3*B*). We then examined chondrogenic ability using pathological staining. Safranine O and Alcian blue staining revealed that the overexpression of miR146a in MSCs by mimic 146a led to decreased cartilage formation compared to that in MSCs treated with mimic NC (Fig. 3, *C* and *D*). Fourteen days after the induction of chondrocytes, MSCs treated with mimic 146a showed reduced COL2A1 and enhanced MMP13 expression as examined by qPCR (Fig. 3, *E* and *F*), indicating that the overexpression of miR146a in MSCs impaired chondrogenesis in vitro.

### MiR146a Directly Targets Lsm11 and Regulates the Expression of Lsm11

**Figure 4.**
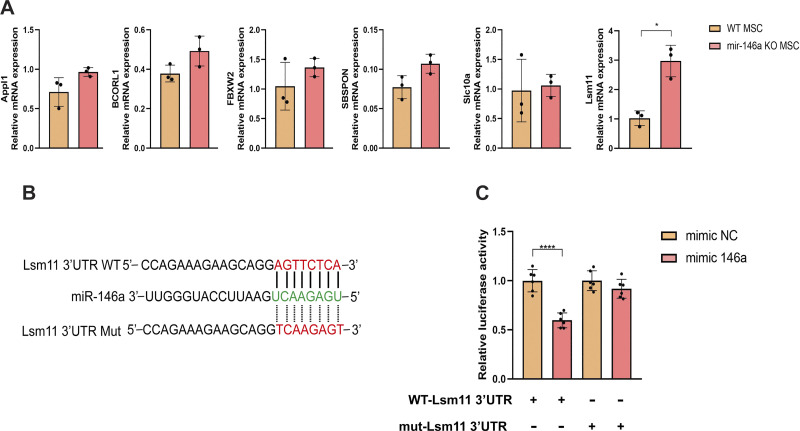
MiR146a targets Lsm11 by directly binding to the region of 3′-untranslated region (UTR). *A*: relative mRNA expression of Appl1, BCORL1, FBXW2, SBSPON, Slc10a, and Lsm11 in wild-type (WT) and miR146a knockout (miR146aKO) mesenchymal stem cells (MSCs) determined by quantitative PCR. *B*: the sequence of the putative miR146a binding site within the 3′-UTR of Lsm11 mRNA. *C*: the WT Lsm11 3′-UTR (WT-Lsm11) reporter plasmid or mutant Lsm11 3′-UTR (mut-Lsm11) reporter plasmid was cotransfected with miR146a-negative control (NC) or miR146a-mimic into HEK293T cells. *P < 0.05, ****P < 0.0001.

We confirmed the modulatory role of miR146a in the chondrogenic process of murine MSCs; however, the exact mechanism of targeting remains unclear. To further clarify the molecular mechanisms, we used the predictive bioinformatics programs TargetScan (http://www.targetscan.org) and miRDB (http://www.mirdb.org/cgi-bin/search.cgi) to predict putative miR146a binding sites in the 3′-UTR. We selected a few potential miR146a-targeted genes at the intersection of different programs with the 3′-UTR binding sites of miR146a and examined their expression by qPCR. The expression of Lsm11, a U7 snRNP-specific Sm-like protein, significantly increased in miR146KO MSCs (Fig. 4*A*). Moreover, we revealed that the 3′-UTR of Lsm11 contained a potential miR146a-binding site (Fig. 4*B*). We then constructed luciferase reporter plasmids containing WT (WT-Lsm11 3′-UTR) or mutant (mut-Lsm11 3′-UTR) Lsm11 binding sites and transfected them into HEK293T cells (Fig. 4*B*). The luciferase activity decreased in 293 T cells in response to miR146a mimic when transfected with plasmids containing WT-Lsm11 binding sites, whereas there were no significant differences when transfected with plasmids with mut-Lsm11 binding sites (Fig. 4*C*), indicating that miR146a inhibits the expression of Lsm11 by binding to the 3′-UTR site of Lsm11.

### MiR146a Regulated Chondrogenesis of MSCs by Targeting Lsm11 In Vivo and In Vitro

**Figure 5.**
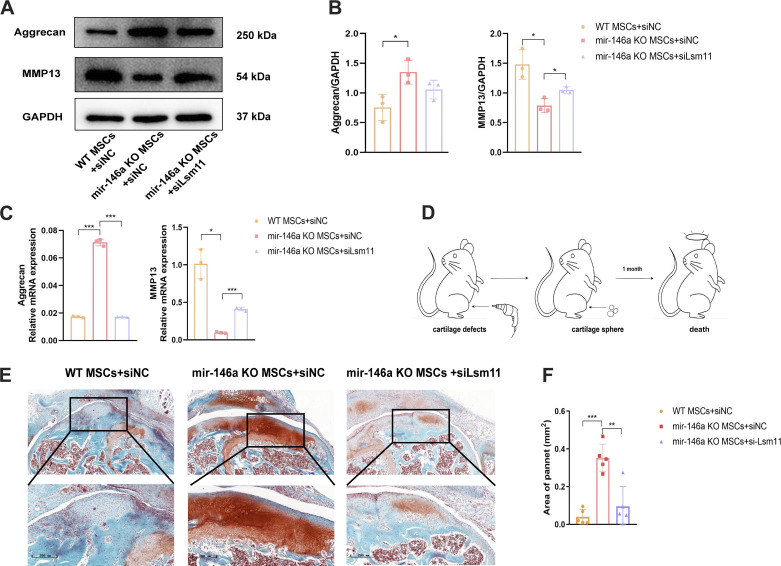
Inhibition of Lsm11 reversed the enhanced ability of chondrogenesis of miR146a knockout (miR146aKO) mesenchymal stem cells (MSCs). *A*: protein expression of aggrecan and matrix metallopeptidase 13 (MMP13) at 14 days after the induction of chondrocytes from wild-type (WT) MSCs treated with siLsm11 negative control (siNC), mir146aKO MSCs treated with siNC, and mir146aKO MSCs treated with siLsm11. *B*: quantification of the protein levels of aggrecan and MMP13 by ImageJ. *C*: relative mRNA expression levels of aggrecan and MMP13 at 14 days after the induction of chondrocytes from WT MSCs treated with siNC, mir146aKO MSCs treated with siNC, and mir146aKO MSCs treated with siLsm11. *D*: isometric cartilage defects of knee joints of 4-mo-old mice were induced by drill penetration at the location of the trochlea of the femur. Cartilage spheres induced from MSCs for 21 days were transplanted to the defect to examine the ability of chondrogenesis and cartilage repair in vivo. Knee joints were collected 1 mo after transplantation of the cartilage spheres (*n* = 6 in each group). *E*: representative images of safranine O staining of cartilage repair by cartilage spheres induced from WT MSCs treated with siNC, miR146KO MSCs treated with siNC, and miR146aKO MSCs treated with siLsm11. Scale bar = 200 µm. *F*: quantification of the area of spheres on the cross section. *P < 0.05, **P < 0.01, ***P < 0.001.

To further evaluate the regulatory effects of Lsm11 on chondrogenesis, we generated siRNA targeting Lsm11 (siLsm11) to inhibit Lsm11 specifically in MSCs in vitro. MSCs from WT and miR146aKO mice were cultured and induced into chondrocytes after treatment with siLsm11 NC (siNC) in vitro. Another group of MSCs from miR146aKO mice was treated with siLsm11 and then cultured and induced to the cartilage sphere to examine whether the inhibition of Lsm11 could block the effect of miR146a knockout in chondrogenesis. After 14 days of induction, the protein and mRNA levels of MSCs were collected to examine their chondrogenic ability in vitro. Knockout of miR146a, as assessed by Western blotting, led to enhanced aggrecan expression and reduced MMP13 expression, whereas treatment with siLsm11 increased the MMP13 level in miR146aKO MSCs compared to that in miR146aKO MSCs treated with siNC (Fig. 5, *A* and *B*). The mRNA level at 14 days after the chondrogenic induction of MSCs also revealed that the inhibition of Lsm11 by siRNA in the miR146aKO MSCs significantly reduced the chondrogenesis compared to that in miR146aKO MSCs treated with siNC, as examined by qPCR of aggrecan and MMP13 (Fig. 5*C*). These results confirmed that miR146a inhibited chondrogenesis by targeting Lsm11 in vitro.

We next proved this result in vivo to determine whether the influence of miR146a and Lsm11 could affect the treatment of cartilage defects, hoping to provide further clinical choices for MSC transplantation in OA interventions. Cartilage defects were generated in mouse joints by drill penetration at the surface of the trochlea of the femur, and cartilage spheres were injected manually at the defective lesion for treatment. One month after injection, the knee joints were collected after mice were euthanized and examined via pathological sections (Fig. 5*D*). Spheres induced from miR146aKO MSCs treated with siNC showed significantly enhanced chondrogenic ability compared to that in the WT MSCs treated with siNC, whereas the inhibition of Lsm11 by siLsm11 remarkably reversed the effect of miR146a knockout on chondrogenesis and showed reduced cartilage repair compared with that in the miR146aKO MSCs treated with siNC, as examined by safranine O staining (Fig. 5*E*), indicating that inhibition of Lsm11 partly blocked the chondrogenic effect of miR146a knockout in MSCs. Areas of different spheres of mice were quantified by safranine O-positive sites in the cross section (Fig. 5*F*). Immunohistochemistry of MMP13 and aggrecan in the tissue also proved the above suggestions (Supplementary Fig. S1; all supplemental material is available at https://doi.org/10.6084/m9.figshare.21904980). In conclusion, both in vivo and in vitro experiments suggest that miR146 inhibits MSC chondrogenesis by targeting Lsm11.

## DISCUSSION

OA is a common degenerative joint disease, and cartilage defects, which are progressive and irreversible, are one of the most obvious pathological features (15). In 2019, it was estimated that OA affected more than 500 million people worldwide and was responsible for 2% of the total global years lived with disabilities (16). The pathophysiology of OA involves the whole joint, including alteration of the composition and structure of the cartilage and chondrocytes, damage of the periarticular bone, abnormal bone remodeling, hyperplasia of the synovial membrane with immune cell infiltration, adipose tissue secreting proinflammatory factors, and mechanical factors (1). Pain relief is the primary therapeutic strategy employed in OA management with surgical joint replacement, the only option at the end stage of disease progression. Early detection and intervention remain unsatisfactory, and there are no disease-modifying treatments for OA. Until 2021, the Osteoarthritis Research Society International and European Society for Clinical and Economic Aspects of Osteoporosis and Osteoarthritis guidelines both recommended key treatments, such as exercise, weight loss, and education, which are conservative for OA management (17). It is believed that pain-modifying treatments will still be essential in the future, as disease-modifying treatments are not yet available (18). For end-stage OA, surgeries are referred for severely affected function (19), including joint replacement surgery, knee osteotomy, knee joint distraction, and arthroscopic knee surgery (18). Early stage OA cannot currently be reversed or cured, partly because of the restricted regeneration of chondrocytes. Therefore, it is essential to investigate the molecular mechanisms of OA and enhance cartilage regeneration through alternative management.

Here, we focused on the regulation of MSCs, which have been widely investigated and used in clinical practice to treat OA because of their remarkable chondrogenic ability. In OA progression, local-resident MSCs in joints undergo loss of function due to aging or disease factors, such as deficiency of proliferation and differentiation and senescence (20, 21). Thus, the injection of MSCs could supply normally functional stem cells as a direct approach for the repair of cartilage defects, or a putative alteration of conventional therapy for OA. MSC treatment of OA is beneficial mainly owing to its potent regeneration and immunomodulation activities, which are essential for cartilage repair (22). The sources of MSCs include adipose tissue, bone marrow, the umbilical cord, and induced pluripotent stem cells (22). In a series of preclinical studies, local delivery of MSCs ex vivo has proven effective against OA owing to the excellent chondrogenic and paracrine effects (23–25), which is a predictive therapy for OA. Thus, for better clinical outcomes of MSC injection, we aimed to investigate the molecules that modulate MSC chondrogenesis.

In this study, we demonstrated that miR146a is a critical regulatory factor in modulating the chondrogenesis of MSCs by directly targeting Lsm11, which might be a novel target for improving the therapeutic effects of MSC transplantation to treat OA. MiR146a is a typical small RNA essential for modulating inflammation and age-related diseases (10, 26). In our previous study, we demonstrated that miR146a is a negative regulator of proinflammatory cytokines in OA and knockdown of miR146a alleviated OA symptoms in mice by regulating cartilage homeostasis (9). Furthermore, miR146a might also be a potential modulator of MSC, which inhibits MSC proliferation partly by interacting with lncRNA (27). These results suggest a complex role for miR146a in the regulation of OA. Here, we aimed to define the mechanisms of miR146a in the chondrogenesis of MSCs, which might elucidate the functions of miR146a in the process of OA. We first found that the expression of miR146a increased during the chondrogenesis of murine MSCs, indicating the potential function of miR146a in regulating chondrogenesis. To further investigate these mechanisms, we generated miR146a homozygous knockout mice, isolated bone marrow MSCs, and cultured them in vitro. We then demonstrated that miR146aKO in MSCs led to the increased formation of chondrocytes, as evidenced by enhanced mRNA expression levels of COL2A1, aggrecan, and COMP and decreased mRNA expression of MMP13 compared to those in WT MSCs, assessed at different time points of the chondrogenic process. In addition, safranine O and Alcian blue staining indicated enhanced chondrogenesis following miR146a deletion. To confirm this result, we successfully overexpressed miR146a in WT MSCs in vitro to determine whether the upregulation of miR146a has the opposite effect on chondrogenesis. In contrast, the overexpression of miR146a by mimic 146a resulted in decreased chondrogenesis, as examined by qPCR and pathological staining throughout the induction process, in which the cartilage sphere induced from MSCs treated with mimic 146a hardly showed the complete shape of the sphere.

These results confirmed that miR146a negatively controls the chondrogenesis process in murine MSCs, but the exact mechanisms have not been elucidated. Based on the mechanisms of microRNA, we aimed to find potential targets for binding to the 3′-UTR of specific genes, which might be a novel regulatory factor of chondrogenesis. Thus we further predicted the targets of miR146a using a database and verified the mRNA expression of potential target genes, in which Lsm11 showed a valid increase in expression in miR146aKO MSCs compared with WT MSCs. Lsm11 is an Sm-like protein in the structure of U7 snRNP that is involved in normal histone pre-mRNA processing by recruiting appropriate cleavage factors; it is also a critical factor for development in *Drosophila* (13). U7 snRNP is composed of U7 snRNA and seven Sm proteins, among which Lsm11 is much larger and different than other Sm proteins because of the extended NH_2_-terminal region, which is important for histone pre-mRNA processing, with the function of replacing the SmD2 protein (28). In addition, a mutation in LSM11 was found in type I interferonopathy, in which the cytosolic DNA sensor cyclic-GMP-AMP synthase-stimulator of interferon genes (STING) pathway was enhanced (29), indicating that Lsm11 correlated with the STING pathway, which could drive the NF-κB signaling pathway and exacerbate OA (30, 31). However, the correlation between Lsm11 and chondrogenesis has not yet been investigated. Thus we aimed to confirm whether alterations in chondrogenesis are correlated with the regulation of Lsm11 by miR146a. We transfected Lsm11 siRNA into miR146aKO MSCs and induced them to chondrocytes in serum-free medium in vitro, which limited the proliferation of MSCs and contributed to the inhibitory effects of siLsm11. SiNC was used in the control groups to minimize the influence of siRNA itself. We observed a significant reduction in chondrogenesis in miR146aKO MSCs treated with siLsm11 compared to those treated with siNC in vivo through the safranine O staining of spheres and in vitro examination by Western blotting and qPCR of ACAN and MMP13. These results indicated that Lsm11 is a direct target of miR146a and promotes chondrogenesis in murine MSCs.

This study had some limitations. For example, the mechanisms by which Lsm11 modulates the chondrogenesis of MSCs remain under investigation, and in future studies, verification of the histone modification of chondrogenic-related mRNA by Lsm11 might explain these phenotypes.

### Conclusions

We demonstrate that miR146a inhibits MSC chondrogenesis by directly targeting Lsm11 and that cartilage spheres derived from miR146aKO MSCs could exert excellent cartilage repair in vivo. Our data suggest that miR146a is a potential target in MSC-based OA therapy.

## DATA AVAILABILITY

Data will be made available upon reasonable request.

## GRANTS

This work was supported by grants from The Ministry of Science and Technology of China (2020YFC2002800), National Natural Science Foundation of China (81830078), Health Commission of Shanghai Municipality (2022JC029), and major research and development project of Shanxi province (201903D321097).

## DISCLOSURES

No conflicts of interest, financial or otherwise, are declared by the authors.

## AUTHOR CONTRIBUTIONS

Y. Liu, Xudong Zhang, X.C., B.Z., L.D., Y. Li, and Xiaoling Zhang conceived and designed research; Y. Liu, x.Z., B.Z., and Xiaoling Zhang performed experiments; Y. Liu, Xudong Zhang., X.C., B.Z., Y. Li, and X.Z. analyzed data; Y. Liu, Xudong Zhang, X.C., L.D., Y. Li, and Xiaoling Zhang interpreted results of experiments; Y. Liu, Xudong Zhang, and B.Z. prepared figures; Y. Lui, Xudong Zhang, and Xiaoling Zhang. drafted manuscript; Y. Lui, Xudong Zhang, X.C., B.Z., L.D., C.W., Y. Li, and Xiaoling Zhang edited and revised manuscript; Y. Lui, Xudong Zhang, X.C., B.Z., L.D., C.W., Y. Li, and Xiaoling Zhang approved final version of manuscript.
